# Medical education in obstetrics and gynecology: A global update from 2025

**DOI:** 10.1111/aogs.70105

**Published:** 2025-11-25

**Authors:** Florian Recker, Ricarda Neubauer, Jana Adams, Sebastian Ludwig, Florin‐Andrei Taran, Tanja Groten

**Affiliations:** ^1^ Department of Obstetrics, Faculty of Medicine and University Hospital Cologne University of Cologne Cologne Germany; ^2^ Department of Gynecology, Faculty of Medicine and University Hospital Cologne University of Cologne Cologne Germany

**Keywords:** competency‐based training, digital health, education, gynecology, medical education, obstetrics, simulation, training

## Abstract

As medical knowledge and technologies rapidly evolve, curricula have become increasingly dense, and designing effective OB‐GYN education that prepares learners for diverse medical careers within limited timeframes is a global challenge. This review provides an international overview of contemporary medical education in obstetrics and gynecology (OB‐GYN) across undergraduate, postgraduate, and continuing professional development levels. A narrative review of recent peer‐reviewed literature, international guidelines, and global initiatives (2023–2025) was conducted, identifying key innovations, trends, and challenges in OB‐GYN education worldwide, with a focus on curriculum reforms, competency‐based education, simulation, telemedicine, AI applications, global standardization, and equity‐oriented initiatives. Undergraduate OB‐GYN curricula are increasingly standardized, integrating core competencies, early clinical exposure, and reproductive health. Postgraduate training adopts competency‐based frameworks, enhanced by simulation, virtual reality, and tele‐education, while continuing medical education has shifted toward flexible digital platforms and structured credentialing. Innovations, such as AI‐driven learning tools, simulation drills, and telemedicine‐based training, have improved skill acquisition, and global bodies, such as FIGO, RCOG, and ACOG, promote curriculum harmonization and equity. The COVID‐19 pandemic accelerated digital adoption but revealed gaps in surgical training and support. Overall, OB‐GYN education is in a transformative phase, marked by technology, standardization, and equity, yet significant disparities persist, especially in resource‐limited settings. Continued global collaboration, investment in educational infrastructure, and adaptive curriculum development are essential to prepare OB‐GYN professionals for evolving clinical demands and healthcare inequities in the postpandemic era.

## Graphical abstract

OB‐GYN education is undergoing a global transformation driven by simulation, competency‐based training, and digital innovations, while international standards aim to reduce persistent resource gaps and improve equitable learning.

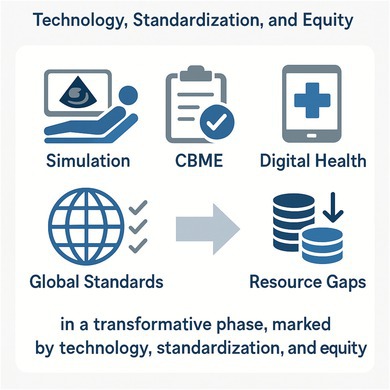

**Abbreviations**
ACOG: American College of Obstetricians and Gynecologists
CBME: Competency‐Based Medical Education
CME: Continuing Medical Education
FIGO: International Federation of Gynecology and Obstetrics
HICs: high‐income countries
LMICs: low‐ and middle‐income countries
OB‐GYN: obstetrics and gynecology
RCOG: Royal College of Obstetricians and Gynecologists

**Key Message**

OB‐GYN education worldwide is rapidly evolving, integrating simulation, digital health, and equity‐oriented frameworks. Sustained global collaboration and competency‐based curricula are essential to prepare future specialists for advancing technologies and diverse women's health needs.

## 1 INTRODUCTION

Medical education in obstetrics and gynecology (OB‐GYN) is pivotal for preparing physicians to address women's health needs across the globe. High‐quality training in this field directly impacts maternal and neonatal outcomes, reproductive health services, and the overall well‐being of women and families. Yet, educational approaches in OB‐GYN are continually evolving in response to emerging medical knowledge, technological advancements, and societal changes. OB‐GYN education is structured across multiple levels—from medical school through residency to subspecialization—each stage requiring the progressive acquisition of hands‐on skills. Examiner‐dependent procedures such as pelvic exams, ultrasound, and obstetric maneuvers demand repeated, supervised training. Yet many students report limited preparation.^1^ Also, the aftermath of the COVID‐19 pandemic has further accelerated changes in how obstetric and gynecological training is delivered, revealing both vulnerabilities and opportunities in existing educational frameworks.^2^, ^3^, ^4^ Major innovations and pedagogical strategies have gained prominence in recent years, including simulation‐based training, the integration of telemedicine into teaching, and emerging applications of artificial intelligence (AI) in medical education. Curriculum reforms, such as the adoption of competency‐based education, along with efforts by leading organizations—such as the International Federation of Gynecology and Obstetrics (FIGO), the Royal College of Obstetricians and Gynecologists (RCOG), and the American College of Obstetricians and Gynecologists (ACOG)—were made to establish global standards.^5^ Nevertheless, disparities in health access and medical resources between high‐income countries (HICs) and low‐ and middle‐income countries (LMICs) as well as the incorporation of equity‐focused initiatives—addressing gender, racial, and socioeconomic disparities—into OB‐GYN training pose major challenges in modern OB‐GYN training.

In recent years, the landscape of OB‐GYN education has been shaped by several transformative trends: a shift toward competency‐based curricula, integration of simulation and digital technologies, efforts to standardize core competencies globally, and initiatives to promote equity and inclusion in training programs.^6^ Interprofessional learning is increasingly emphasized, especially through joint simulations with midwives and nurses. Team‐based obstetric emergency training has been linked to lower neonatal morbidity and improved communication and preparedness.^7^, ^8^

The aim of this article is to provide a comprehensive global update on medical education in OB‐GYN, encompassing the full spectrum from undergraduate medical education through postgraduate specialty training.

## 2 MATERIAL AND METHODS

**FIGURE 1:**
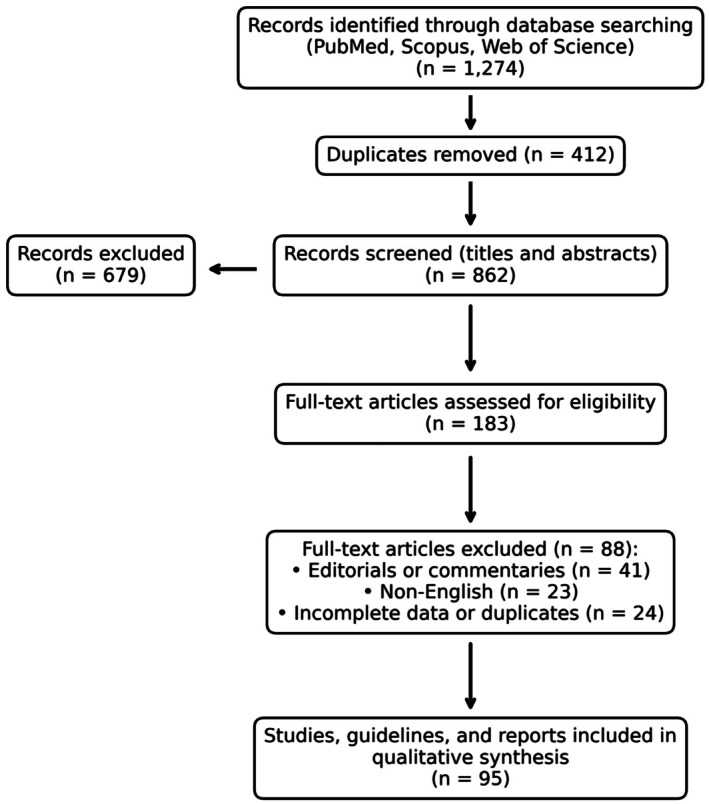
Flow of records through the literature search and selection process across PubMed, Scopus, and Web of Science databases for studies addressing global trends in OB‐GYN medical education (2023–2025).

The search was conducted across PubMed, Scopus, and Web of Science using the following Boolean string: (“obstetrics and gynecology” OR “OB‐GYN”) AND (“education” OR “training” OR “curriculum”) AND (“simulation” OR “telemedicine” OR “competency‐based” OR “AI” OR “digital health”) AND (2023:2025[dp]). Titles and abstracts were screened for relevance, followed by full‐text evaluation. Inclusion criteria comprised peer‐reviewed articles, guidelines, or global initiatives addressing educational frameworks or innovations in OB‐GYN. Exclusion criteria were editorials without data, non‐English publications, and duplicated conference abstracts. The selection process followed PRISMA principles and is illustrated in Figure 1.

Sources included peer‐reviewed journal articles (retrieved via databases such as PubMed), consensus guidelines and curriculum documents from professional bodies (e.g. FIGO, RCOG, ACOG), and reports from international initiatives focused on OB‐GYN training. Relevant publications were screened for inclusion based on relevance to one or more of the following domains: undergraduate medical education curriculum in OB‐GYN during medical school, postgraduate training during residency or specialist training in OB‐GYN, CME or professional development for OB‐GYN practitioners, educational innovations, global or regional comparisons, and equity or diversity initiatives in OB‐GYN training. Data from included sources were extracted and thematically organized. Given the breadth of topics, no formal quantitative meta‐analysis was performed; instead, a narrative approach was chosen for data synthesis.

## 3 RESULTS

### 3.1 Undergraduate Education in OB‐GYN

Undergraduate medical education serves as the foundation for future OB‐GYN specialists, and recent reforms have focused on ensuring that all medical graduates attain core competencies in women's health. Modern medical schools worldwide are increasingly adopting integrated curricula that weave obstetric and gynecological knowledge throughout the medical program, rather than confining it to a single clerkship.^9^ This integration often places OB‐GYN teaching alongside related basic sciences and other clinical disciplines, helping students contextualize reproductive health concepts within overall patient care.^9^, ^10^ Innovative pedagogical methods such as problem‐based learning (PBL) and team‐based learning (TBL) are being used to enhance student engagement and critical thinking in OB‐GYN topics.^11^ These student‐centered approaches encourage the application of theoretical knowledge to clinical scenarios, preparing learners for the dynamic and unpredictable nature of obstetric and gynecologic practice.

Recognizing variability in medical school OB‐GYN training across institutions and countries, international efforts have been made to define a “common curriculum” for OB‐GYN. In 2024, an international FIGO taskforce published recommendations for a core OB‐GYN curriculum for medical students globally.^5^ This proposed global curriculum delineates essential learning objectives across three domains—clinical skills, professional behaviors, and knowledge—covering conditions affecting women in diverse social and cultural contexts.^5^ The intent is to standardize the minimum competencies of new medical graduates in OB‐GYN, ensuring they can provide basic women's health care and address important issues such as family planning, safe motherhood, and gynecologic screenings regardless of where they are trained.^5^ For instance, the curriculum emphasizes proficiency in taking gynecologic history and pelvic examinations, understanding contraception and sexually transmitted infection prevention, recognizing obstetric emergencies, and exhibiting respectful, ethical patient care. By establishing global benchmarks, this effort aims to improve women's health outcomes by leveling up training quality worldwide.^5^ Ongoing work is looking at how this core curriculum might be adapted in special circumstances (e.g. condensed “minimum” curricula during crises like pandemics or conflicts).^5^

A notable undergraduate curriculum development has been the push to incorporate comprehensive sexual and reproductive health and rights (SRHR) education – including contraception and safe abortion care – into all medical school programs. The Association of Professors of Gynecology and Obstetrics (APGO) recommends including pregnancy options counseling as a standard component of the pre‐clinical undergraduate medical curriculum.^12^ A joint statement in 2022 by FIGO, the International Federation of Medical Students' Associations (IFMSA), and the World Association of Trainees in OB‐GYN (WATOG) called for universal inclusion of SRHR topics in undergraduate medical training.^13^ This “gold standard” curriculum framework emphasizes that medical students should receive education in contraception and abortion care spanning basic sciences, clinical skills, public health, ethics, and human rights.^13^ The rationale is that early‐career training in these areas is essential to produce physicians who can meet women's basic health needs, especially given global concerns about shortages of providers trained in abortion care and related services.^13^ Medical student advocacy has been a driving force in this area: surveys indicate that students in many countries perceive gaps in their OB‐GYN curriculum regarding family planning and desire more training in these topics.^13^ Although a majority of respondents viewed SRHR education as important (88%) and feasible (76%), commonly reported barriers included cultural sensitivity of topics, lack of faculty support, curriculum overload, and dependence on tuition income and institutional rankings.^14^

In response, some medical schools (often with support from national OB‐GYN associations) have started to introduce dedicated modules on SRHR, partner with family planning organizations for clinical exposure, and normalize the teaching of sensitive topics like pregnancy options counseling. These efforts reflect a broader trend toward aligning medical education with public health priorities and human rights standards in women's health care. Medical students themselves are also driving such collaborations. The international organization Medical Students for Choice (MSFC) offers targeted programs to facilitate clinical exposure to family planning.^15^ For instance, MSFC allocates funds to support a Reproductive Health Externship program, which enables students to engage in a minimum of 2 weeks of internship experience at a designated abortion clinic of their choosing. Furthermore, MSFC has established unique collaborative relationships, such as its partnership with the British Pregnancy Advisory Service (BPAS) in the United Kingdom, which facilitates 1‐week internships, and offers 2‐week training placements at a regional hospital in Uganda.^16^

Undergraduate OB‐GYN education is also benefiting from the broader trend of experiential and simulation‐based learning. Many medical schools now supplement clinical rotations in OB‐GYN with simulation exercises to help students practice basic skills in a safe environment.^17^ For example, simulation models and standardized patients are used to teach pelvic or breast examinations, labor and delivery maneuvers, and emergency response to medical students.^18^, ^19^ A recent study demonstrated that an obstetric simulation curriculum combined with online modules significantly improved medical students' knowledge and confidence in managing obstetric scenarios.^20^, ^21^ High‐fidelity childbirth simulators and virtual reality scenarios are increasingly accessible in teaching hospitals, allowing students early hands‐on practice without risk to patients. Additionally, interactive e‐learning platforms have been developed to reinforce OB‐GYN topics; students can engage in virtual case discussions or hone skills like fetal heart tracing interpretation via online tools. These approaches, introduced at the undergraduate level, not only enhance learning outcomes but also stimulate student interest in OB‐GYN careers by providing a more engaging experience.^10^, ^22^ Indeed, exposure to well‐designed OB‐GYN curricula and positive role models during medical school has been linked to a greater likelihood of students choosing OB‐GYN for residency.^10^ As workforce shortages loom in some regions, enriching the undergraduate OB‐GYN experience is seen as a strategy to attract the next generation to this field.

With competency‐based education gaining traction, medical schools are refining how they assess students in OB‐GYN. Instead of relying solely on traditional knowledge exams, many programs now use objective structured clinical examinations (OSCEs) featuring OB‐GYN stations. Entrustable Professional Activities (EPAs)—such as “performing a normal vaginal delivery” or “counseling a patient on prenatal screening”—are being defined for medical students and used as benchmarks for graduation readiness.^23^ The shift toward competency‐based assessment means students must not only know information but also demonstrate core skills and professional behaviors in OB‐GYN before entering residency. This is aligned with the global calls for outcome‐based education ensuring all new graduates have a baseline ability to contribute to women's healthcare.^5^

### 3.2 Postgraduate (residency) training in OB‐GYN

Postgraduate training is where medical graduates refine their skills into specialized clinical competency. OB‐GYN residency (or specialist registrar training) programs worldwide are progressively adopting competency‐based medical education (CBME) frameworks. Traditional time‐based training models (e.g., a fixed 4‐year residency) are being augmented or restructured such that advancement is tied more closely to achieving defined competencies (knowledge, surgical skills, patient management, etc.) rather than just time served. In the United States, the Accreditation Council for Graduate Medical Education (ACGME) has implemented Milestones—a competency‐based evaluation system—for OB‐GYN residents, which was updated in recent years to Milestones 2.0 to better define progressive expertise in areas like obstetric care, gynecologic surgery, and systems‐based practice.^24^ Similarly, the Royal College of Physicians and Surgeons of Canada introduced the Competence by Design initiative, and OB‐GYN residency programs have been transitioning to this staged competency attainment model.^25^ In the United Kingdom, the RCOG rolled out a new curriculum in 2019 emphasizing capabilities in practice (CiP) and entrustable professional activities that residents must meet to attain a Certificate of Completion of Training.^26^ Early evidence suggests that these competency‐driven curricula help identify residents who need additional support in specific areas and ensure a more well‐rounded skill set at graduation, although implementation has not been without challenges (including increased faculty assessment burden and the need for robust assessment tools).

CBME emphasizes outcomes over time‐based training, enhancing transparency, empowering learners and improving patient safety.^27^ A 2025 Delphi study merged existing frameworks to develop a unified OB‐GYN competency framework with 91 competencies, illustrating ongoing efforts to standardize postgraduate training and facilitate international comparisons.

Simulation has become a cornerstone of OB‐GYN residency training across many countries.^9^ Residency programs increasingly use high‐fidelity simulation labs for teaching obstetric emergencies and surgical procedures in a controlled, team‐based setting. Obstetric emergency drills (e.g., managing postpartum hemorrhage, eclampsia, or shoulder dystocia) are often practiced in interprofessional groups with residents, midwives, nurses, and anesthesiology trainees working together. Team‐based simulation training has been shown to improve not only technical management of emergencies but also communication and teamwork, which are critical for patient safety in obstetrics.^28^ One well‐known simulation program is Practical Obstetric Multi‐Professional Training (PROMPT), originally developed in the United Kingdom, which has demonstrated significant improvements in obstetric outcomes when implemented regularly.^28^ A national scale‐up of PROMPT in Wales, for example, trained local multi‐professional facilitators in all units and achieved over 80% staff participation within 15 months.^29^

Surgical simulation is equally vital on the gynecology side of training. Given restricted working hours and patient safety considerations, residents have fewer opportunities to practice complex surgeries on patients. To address this, simulations using anatomical models, virtual reality laparoscopic simulators, and cadaveric or animal tissue labs are utilized for training in operative gynecologic and perinatal procedures.^30^ Competency‐based surgical skill curricula have been implemented in some programs, where residents must demonstrate proficiency on simulators or supervised models (e.g., performing a simulated laparoscopic tubal ligation) before they are entrusted with real cases in the operating room. This ensures a baseline skill level that enhances patient safety. In obstetrics, surgical simulations such as mock cesarean deliveries (including managing uncommon complications like a cesarean with an impacted fetal head or a uterine inversion) provide exposure to scenarios that might not occur frequently during training. A recent nationwide survey of OB‐GYN residents in Portugal highlighted that while common scenarios (normal delivery, vacuum/forceps delivery, breech birth) were widely practiced in simulation by >90% of residents, rare but critical scenarios like cesarean with impacted fetal head, amniotic fluid embolism, and maternal cardiac arrest were underrepresented.^31^ This finding has led to recommendations to broaden the scope of simulation training to cover high‐acuity, low‐frequency events, so that graduates are better prepared for such emergencies.^31^

Postgraduate OB‐GYN training varies considerably worldwide in duration, case mix, and oversight, but there are ongoing efforts to harmonize standards. International collaborations and exchange programs have grown, allowing trainees to rotate abroad or take international board examinations. FIGO has facilitated workshops and curriculum development in LMICs to align local training with global competencies. For example, FIGO's initiative to create a Global Competency‐Based Fistula Surgery Training Manual provides a standardized curriculum to train surgeons in the subspecialty skill of obstetric fistula repair in countries where fistula is prevalent.^32^ Additionally, global consensus statements (like the 2018 Consensus on Residency Training in OB‐GYN by APGO and CREOG in the United States, or the European Board & College of Obstetrics and Gynecology logbook in Europe) share best practices on minimum operative numbers, exposure to subspecialties and assessment methods. Standardized in‐training exams are used to compare knowledge across programs and ensure consistency. As physician migration increases, such efforts aim to ensure that an OB‐GYN specialist trained in one country has the requisite competencies to practice safely in another, aiding global workforce mobility and exchange of expertise.^5^

### 3.3 Continuing medical education and professional development

The pace of advancements in OB‐GYN—from new surgical techniques (e.g., minimally invasive and robotic surgery) to emerging medical therapies (e.g., novel pharmaceuticals for fibroids or infertility)—necessitates that practicing OB‐GYNs continually update their knowledge and skills. CME is thus a critical component of an OB‐GYN's professional life. Many jurisdictions require OB‐GYN specialists to earn a certain number of CME credits per cycle (often annually or biannually) to maintain licensure or certification. For example, the American Board of Obstetrics and Gynecology (ABOG) maintenance of certification program mandates ongoing learning and periodic assessments.^33^

Even prior to the pandemic, online CME was growing, but recent years have cemented its central role. OB‐GYN professionals now routinely engage in webinars, virtual conferences, and online modules to fulfill their learning needs.^9^ The convenience of digital CME—allowing clinicians to learn at their own pace and schedule—has dramatically increased participation. For instance, major annual conferences such as the FIGO World Congress or RCOG World Congress pivoted to virtual or hybrid formats during the pandemic, enabling thousands of participants globally to attend sessions online. This has democratized access to cutting‐edge updates; an obstetrician in a remote LMIC setting can now virtually attend lectures by international experts that previously required costly travel. Professional bodies have also developed sophisticated e‐learning platforms: ACOG's online “APGO Academic Scholars and Leaders” programs and RCOG's StratOG modules are examples of curated online curricula for topics ranging from basic obstetric skills to leadership training.^34^, ^35^ Digital platforms and mobile applications have revolutionized CME, allowing OB‐GYN clinicians to seamlessly integrate learning into their daily routines.^9^ Mobile apps provide on‐the‐go resources that practitioners can consult in clinic or on call, turning downtime into learning opportunities.

Continuing education in OB‐GYN is not limited to lectures and readings. There is increasing recognition that experienced providers benefit from periodic simulation‐based refresher training, particularly for managing obstetric emergencies or infrequent high‐risk events. Many hospitals and health systems now conduct regular drills that involve not just trainees but also attending physicians, nurses, and other staff. These drills serve as continuing training to reinforce best practices and team coordination. Notably, the implementation of programs like PROMPT is intended for all maternity unit staff, meaning even seasoned OB‐GYNs participate alongside residents in simulation exercises.^29^ Evaluation of these programs has shown improvements in patient outcomes and safety culture when the majority of staff are trained in the latest protocols and algorithms.^29^ In gynecology, workshops on new technologies are commonly offered at conferences or regional centers. The concept of “surgical bootcamps” for graduating residents or new specialists is also emerging: intensive short courses that cover key procedures and possible complications through simulation to ensure readiness for independent practice.^31^

Recognizing the need to document and encourage lifelong learning, organizations like FIGO have launched CPD platforms. FIGO's CPD initiative, for example, allows OB‐GYN professionals globally to record their educational activities and earn an FIGO Certificate of Continuing Education.^36^ This not only provides individuals with evidence of their up‐to‐date knowledge but also helps standardize what counts as meaningful education across countries.

### 3.4 Global sharing of knowledge

Recent times have also seen an increase in international collaboration in continuing education. Multi‐center grand rounds held via Zoom, global journal clubs on social media, and collaborative guideline development all serve to keep practitioners informed. An illustrative example is the global dissemination of WHO guidelines through FIGO's cascade training model for family planning: experienced clinicians in various countries were trained on new contraceptive care tools and then tasked with training peers locally, leading to widespread uptake of updated practices and even influencing national policy.^37^ Such initiatives blur the line between formal CME and health system strengthening, highlighting that continuing education can drive system‐level improvements.

### 3.5 Innovations and technological trends in OB‐GYN education

Rapid technological progress and pedagogical innovation have profoundly influenced how OB‐GYN education is delivered at all levels. In the following, some of the major innovations and trends reshaping training are discussed.

The use of simulation in OB‐GYN training has expanded from simple task trainers to sophisticated high‐fidelity simulators and virtual environments. High‐fidelity mannequins that simulate childbirth (complete with physiological responses like bleeding or fetal heart rate changes) are now common in teaching hospitals, enabling realistic practice of deliveries, shoulder dystocia maneuvers, postpartum hemorrhage management, and neonatal resuscitation. Team‐based simulation drills not only teach technical skills but also improve crew resource management during emergencies. Virtual reality and augmented reality are emerging as next‐generation tools: for example, VR modules allow learners to perform a complete hysteroscopic procedure or navigate a complicated delivery in a fully virtual setting, while AR can overlay guidance or anatomy on a live view to teach ultrasound or surgical anatomy.^9^ These immersive technologies have been shown to enhance learner engagement and knowledge retention by simulating complex OB‐GYN procedures without patient risk. A key advantage is the ability to repeat scenarios multiple times until mastery is achieved. As costs of VR/AR come down, more programs in both high‐ and middle‐income countries are piloting their use for both students and residents.

AI applications in medical education are nascent but growing. AI‐driven tools can personalize learning by adjusting content difficulty based on learner performance, identify knowledge gaps, and even generate practice questions or cases tailored to the trainee's needs. In OB‐GYN, one area of exploration is AI‐powered virtual patients: computer programs that present lifelike obstetric or gynecologic case scenarios and respond to student inputs, providing feedback and tutoring. Additionally, AI is being used to provide real‐time feedback in simulations—such as an AI algorithm that analyzes a resident's ultrasound technique or surgical movements and immediately points out errors or areas to improve. AI‐enhanced simulations can significantly aid OB‐GYN residents in refining technical skills by tracking performance and offering objective critiques.^38^ Another AI contribution is in knowledge management: AI‐driven search and summary tools can help trainees stay up‐to‐date by quickly summarizing new research or guidelines, acting as a smart study assistant. Large language models have even been tested in generating counseling scripts for patient interactions and providing answers to clinical queries; early studies found that such models can produce competent counseling templates for OB‐GYN scenarios.^39^, ^40^ While AI holds promise—from intelligent tutoring systems to automated skills assessment—educators caution about the need to validate these tools and ensure trainees continue to learn critical thinking, not just accept AI suggestions. Nevertheless, careful integration of AI in training could augment traditional teaching, offering more personalized, data‐driven educational experiences for OB‐GYN learners.^38^

Innovative assessment methods are being deployed to support competency‐based education. Electronic logbooks and portfolios allow detailed tracking of procedures performed and competencies achieved by each trainee. Some programs use smartphone apps where residents log their operative cases and get immediate feedback from supervisors who also record entrustment decisions (e.g., “allowed to do independently” vs. “required significant help”).

Given the central role of ultrasonography in OB‐GYN, there have been particular endeavors to innovate ultrasound education. The concept of competency‐based ultrasound education involves using simulators (like ultrasound training mannequins or VR simulators) and objective metrics to train learners until they demonstrate proficiency.^9^

### 3.6 Global and regional perspectives: high‐income vs. low‐resource settings

A global update on OB‐GYN education must recognize the disparate contexts in which training occurs. There are marked differences between HICs and LMICs in terms of educational infrastructure, clinical exposure, and challenges faced. The training infrastructure, staffing, curricula, use of technology, clinical experience opportunities, and work‐related challenges are often fundamentally different. In HIC, training facilities are typically well developed with access to modern simulation centers, stable internet connectivity and numerous teaching hospitals. It is encouraging to note that a significant number of important educational innovations tailored to the needs of LMICs have been implemented in these countries. In several African countries, mid‐level providers have been trained via simulation to perform lifesaving interventions (like manual vacuum aspiration for miscarriage care or assisted deliveries), effectively expanding the pool of skilled providers. Another example is the “cascade” model of faculty development—a successful FIGO‐WHO collaboration trained regional master trainers in family planning who then trained thousands of healthcare workers in multiple countries, resulting in sustained updates to curricula and practice norms.^37^ The outcome of such programs often includes policy changes (e.g., health ministries mandating inclusion of certain skills in training), showing how education can drive system reform. Furthermore, limited connectivity and infrastructure deficits in LMICs make modern teaching and exchange difficult. On top of that, the number of training institutions is limited. In 2011, it was estimated that there were only approximately 250 OB‐GYN postgraduate training positions across Africa for just under 1 billion people.^41^ This is a fraction of what is needed to meet demand. This shortage of local training opportunities is a significant factor contributing to the “brain drain”.^42^ As a result, highly qualified obstetricians and gynecologists often migrate from LMICs to richer countries, which benefit from the training costs invested in LMICs.^43^ In most cases, HICs have implemented standardized, competency‐based training programs lasting 4–6 years, often with dedicated rotations in subspecialties (e.g., MFM, gyn oncology).

### 3.7 Telemedicine, tele‐education, and the impact of COVID‐19 ON OB‐GYN training

Postgraduate OB‐GYN training has traditionally relied on in‐person, hands‐on clinical experience. However, the global integration of telemedicine, accelerated by the COVID‐19 pandemic, has profoundly influenced both the structure and delivery of residency education. When outpatient services were rapidly converted to telehealth formats during 2020, many clinics transitioned prenatal visits, contraception counseling, and postoperative follow‐ups to virtual platforms.^2^ Residents and fellows suddenly found themselves conducting or co‐leading telemedicine consultations, often without prior formal training. This experience exposed the need for explicit instruction in telemedicine competencies—including effective video communication, coaching patients in self‐administered assessments (such as blood pressure or fundal height measurement), and adapting sensitive counseling to a virtual format.

Professional organizations such as ACOG and the American Medical Association subsequently issued guidance recommending that residency programs integrate telehealth objectives into core curricula.^2^ These competencies include mastery of “webside manner,” awareness of regulatory and privacy frameworks, and the ability to conduct hybrid clinical encounters that combine remote and in‐person components. In practice, many residents now experience blended outpatient rotations where telehealth clinics complement traditional in‐person sessions under direct or virtual supervision. Beyond clinical care, telemedicine has become an educational tool itself: didactic lectures, case conferences, and simulation‐based teaching are now routinely delivered via secure video platforms, a model first adopted during pandemic lockdowns and largely retained thereafter.

The necessity of remote clinical supervision further normalized the use of tele‐mentoring and digital collaboration. Attending physicians increasingly supervised residents via secure video platforms, providing real‐time feedback on consultations or ultrasound exams when physical presence was restricted. At the same time, the shift to virtual formats underscored the importance of wellness and resilience in digital learning environments. Isolation, workload uncertainty, and disrupted clinical exposure contributed to increased stress and burnout among trainees.^3^ In response, many programs incorporated structured wellness modules—covering coping strategies, peer support networks, and mental‐health resources—into their curricula. These measures, alongside hybrid scheduling flexibility, have strengthened the long‐term sustainability of digitally enhanced education.

The rapid expansion of telemedicine also transformed global OB‐GYN education into a fertile field for research. Institutions worldwide evaluated virtual teaching effectiveness, learner satisfaction, and skill retention, generating a new evidence base that continues to inform curriculum reform. International organizations such as FIGO and AOFOG have since endorsed hybrid and tele‐education approaches as integral to postgraduate and continuing medical education. In LMICs, donor‐supported programs now combine virtual instruction with regional simulation workshops to address persistent training inequities and skill gaps.

## 4 DISCUSSION

This global overview of OB‐GYN medical education reveals a field in dynamic evolution, responding to both longstanding challenges and recent paradigm shifts. The integration of simulation, digital tools, and potentially AI, has generally been a success story, enhancing training quality. For example, multiple studies in low‐resource settings have linked obstetric emergency simulation training to reduced perinatal complications and better adherence to protocols. In high‐resource settings, sustained simulation programs correlate with lower obstetric litigation and adverse events. However, challenges remain in scaling these methods sustainably—issues of cost, faculty time, and ensuring simulation fidelity and relevance need ongoing attention. The increasing interest in VR/AR and AI as training adjuncts is promising, but these tools must be rigorously evaluated within OB‐GYN education to establish their added value. There is also an equity consideration: if advanced technologies become the gold standard in training, we must ensure that trainees in resource‐limited environments are not left behind. Creative dissemination (such as open‐source virtual simulation modules or sharing of equipment through partnerships) will be important to democratize these innovations.

The push for globally standardized curricula, exemplified by FIGO's core curriculum initiative for medical students,^5^ speaks to the aspiration of a more uniform foundation in OB‐GYN knowledge and skills worldwide. Standardization can facilitate international collaboration, easier transition of graduates across countries, and a collective rise in competence, but must be balanced with local customization. As noted, OB‐GYN health needs and resource realities differ. HICs grapple with ensuring adequate surgical experience in the face of safer, noninvasive treatments, managing trainee burnout, integrating rapidly evolving technologies into training, and promoting diversity and inclusion within programs.

The infusion of technology and innovative methods in OB‐GYN education serves to enhance learning efficiency, provide safe practice environments, and ultimately improve clinical preparedness of graduates. Importantly, research indicates that even low‐fidelity simulations combined with e‐learning can significantly boost knowledge and skills in OB‐GYN for medical and nursing students.^44^

Additionally, the pandemic underscored that well‐being is a prerequisite for learning. Thus, wellness is now being treated as a quality indicator of training programs representing a cultural shift in graduate medical education from the bygone “endure hardships” mentality to a more humane approach that recognizes limits and the need for support.

Evidence quality across themes varied. Simulation‐based training shows that realistic, risk‐free environments enhance technical and nontechnical skills.^45^ However, many studies are short‐term or descriptive, with limited data on long‐term patient outcomes. Telehealth innovations improve care continuity and reduce burdens,^46^ yet inequities in digital access persist. Competency‐based education enhances transparency and patient safety, but rigorous implementation frameworks are sparse. Digital health competencies frameworks (e.g., DECODE) outline 4 domains, 19 competencies, and 33 mandatory outcomes, emphasizing digital literacy and equity,^47^ yet integrating these into busy curricula remains challenging. SRHR training modules, such as the WHO‐supported program in Benin, empower frontline health workers and employ interactive, problem‐based learning; however, their applicability to other settings requires adaptation. AI integration promises precision care but raises concerns about data bias and clinician trust, underscoring the need for thorough education and clear regulations.^48^

In discussing global OB‐GYN education, this review faces limitations such as potential bias toward English‐language and published literature, which may underrepresent innovative practices from non‐English‐speaking regions or informal knowledge exchange. There is also an imbalance in data availability—much is reported from high‐income settings in the literature, whereas challenges and improvements in LMICs may be underreported except through broader health metrics.

## 5 CONCLUSION

Medical education in OB‐GYN is marked by innovation, adaptability, and a global outlook unprecedented in prior eras. Educational strategies now prioritize hands‐on skills and validated competencies, leveraging simulation and technology to produce clinicians who are both technically adept and deeply cognizant of the humanistic and societal dimensions of women's health care. In conclusion, the global state of OB‐GYN medical education is one of constructive change—integrating new teaching modalities, breaking traditional silos between regions and professions, and committing to a competency‐driven, equity‐oriented training paradigm. By sustaining this momentum and addressing remaining challenges, the OB‐GYN educational community can better prepare the next generation of providers to deliver safe, evidence‐based, and compassionate care, ultimately contributing to improved reproductive health outcomes worldwide.

## AUTHOR CONTRIBUTIONS

FR conceived and designed the study. RN, JA, SL, FAT, and TG contributed to the literature review, data synthesis, and drafting of the manuscript. FR revised the manuscript critically for important intellectual content. All authors read and approved the final version of the manuscript.

## FUNDING INFORMATION

No specific funding was received for this work.

## CONFLICT OF INTEREST STATEMENT

The authors declare that they have no competing interests.

## Data Availability

The data supporting the findings of this study are available from the corresponding author upon reasonable request.
